# Molecular changes in articular cartilage and subchondral bone in the rat anterior cruciate ligament transection and meniscectomized models of osteoarthritis

**DOI:** 10.1186/1471-2474-12-197

**Published:** 2011-08-24

**Authors:** Maureen Pickarski, Tadashi Hayami, Ya Zhuo, Le T Duong

**Affiliations:** 1 Merck Sharpe & Dohme Corp., Bone Biology Group, West Point, PA 19486, USA

**Keywords:** osteoarthritis, subchondral bone, cartilage degeneration, bone remodeling

## Abstract

**Background:**

Osteoarthritis (OA) is a debilitating, progressive joint disease.

**Methods:**

Similar to the disease progression in humans, sequential events of early cartilage degradation, subchondral osteopenia followed by sclerosis, and late osteophyte formation were demonstrated in the anterior cruciate ligament transection (ACLT) or ACLT with partial medial meniscectomy (ACLT + MMx) rat OA models. We describe a reliable and consistent method to examine the time dependent changes in the gene expression profiles in articular cartilage and subchondral bone.

**Results:**

Local regulation of matrix degradation markers was demonstrated by a significant increase in mRNA levels of aggrecanase-1 and MMP-13 as early as the first week post-surgery, and expression remained elevated throughout the 10 week study. Immunohistochemistry confirmed MMP-13 expression in differentiated chondrocytes and synovial fibroblasts at week-2 and cells within osteophytes at week-10 in the surgically-modified-joints. Concomitant increases in chondrocyte differentiation markers, Col IIA and Sox 9, and vascular invasion markers, VEGF and CD31, peaked around week-2 to -4, and returned to Sham levels at later time points in both models. Indeed, VEGF-positive cells were found in the deep articular chondrocytes adjacent to subchondral bone. Osteoclastic bone resorption markers, cathepsin K and TRAP, were also elevated at week-2. Confirming bone resorption is an early local event in OA progression, cathepsin K positive osteoclasts were found invading the articular cartilage from the subchondral region at week 2. This was followed by late disease events, including subchondral sclerosis and osteophyte formation, as demonstrated by the upregulation of the osteoanabolic markers runx2 and osterix, toward week-4 to 6 post-surgery.

**Conclusions:**

In summary, this study demonstrated the temporal and cohesive gene expression changes in articular cartilage and subchondral bone using known markers of OA progression. The findings here support genome-wide profiling efforts to elucidate the sequential and complex regulation of the disease.

## Background

Osteoarthritis (OA) is a joint disease that involves degeneration of cartilage, limited synovitis, subchondral bone changes, and osteophyte formation [1]. Mechanical stress associated with joint instability is thought to induce changes in biochemical factors within affected joints leading to focal articular cartilage degradation [2]. However, recent evidence indicates that it is also necessary to consider the contributions of synovial inflammation and subchondral bone changes [3]. The integrity of the articular cartilage has been proposed to depend on the biomechanical properties of the underlying bone [4]. Subchondral bone changes detected by 99mTc scintigraphy appeared to precede radiographic signs of structural damage in nodal OA joints [5]. More recent studies have documented acceleration of subchondral bone turnover accompanied by specific architectural changes in the subchondral trabecular bone of the OA joints [6-9]. Furthermore, epidemiologic studies have correlated both the increases in bone mineral density and in the rate of bone turnover, as determined by biochemical markers, with increases in the incidence and severity of osteoarthritis [10-12]. Because the subchondral bone is critically important in containing the mechanical abnormalities that damage the cartilage, emphasis on a panel of biomarkers of bone remodeling resulting from the abnormal stresses on the joint has been proposed as diagnostic tools used to monitor treatment responses to potential structure-modifying drugs [13,14]. The mechanical and biochemical properties of the subchondral bone are therefore of particular interest in any attempt to determine the molecular mechanisms responsible for initiating osteoarthritis.

Currently, no OA animal models fully mimic the pathogenesis of the human disease and the surgical models of mechanical instability appear to represent chronic, traumatic OA. Among these models, the anterior cruciate ligament transection model in dogs and the partial menisci resection model in rabbits have been widely used to study the histological and biochemical changes occurring during OA progression [15]. As the metabolism of articular cartilage and particularly of the subchondral bone in these species is not completely understood, further analysis of molecular changes in the joints of these species during disease progression is warranted. We have previously reported the characterization of the morphological and histological changes in cartilage and subchondral bone in two different models of surgically-induced OA in the rat, the anterior cruciate ligament transection (ACLT) model or the ACLT in combination with partial medial meniscectomy (ACLT+MMx) [16]. In both models, OA-related pathogenic changes occurred in a time dependent manner with milder and slower disease progression in ACLT, as compared with ACLT+MMx. Surface cartilage damage and accelerated subchondral bone resorption were observed within 2-weeks post-surgery. Significant cartilage thinning, subchondral sclerosis and osteophyte formation were demonstrated as late stage disease events. Here, we focus on evaluating molecular changes of OA progression in these two surgically-induced models of joint instability in rats in order to further our understanding of the relationship between subchondral bone changes and cartilage degradation.

## Methods

### Osteoarthritis model and treatment

All procedures were carried out in accordance with the Institutional Animal Care and Use Committee in Merck Research Labs. A total of 144 intact male Sprague-Dawley rats (Taconic, NY) were used in three time course studies.

Detailed development and characterization of the osteoarthritis (OA) models were previously reported [16]. In brief, the two OA models were surgically induced in 10 week-old male rat right knee joints with some modifications. Rats were anesthetized with isoflurane. After shaving the right knee joint, the skin was disinfected with iodine. The knee joint was exposed though the medial parapatellar approach. The patella was dislocated laterally and the knee placed in full flexion. The anterior cruciate ligament (ACL) was then transected with micro-scissors; complete transection was confirmed with the anterior drawer test. In the more severe model of OA, anterior cruciate ligament transection (ACLT) with partial medial meniscectomy (ACLT+MMx), the medial collateral ligament was additionally transected with resection of medial meniscus. Following surgery, the joint surface was washed with sterile saline solution, and both capsule and skin were sutured using Vicryl 4-0 (Ethicon, Edinburgh, UK), absorbable suture and monofilament 4-0 Nylon threads (Ethicon, Edinburgh, UK).

In the sham operation, after subluxation of patella with the same procedure as OA model, the wound was closed by layers. All operation procedures were performed using a surgical loupe. Buprenorphine hydrochloride (0.1 mg/kg) (Reckitt & Colman Products Ltd., Hull, England) was given as a post-operative analgesic. Rats were allowed to move freely in the soft bedding in plastic cages.

The initial time-course study followed the animals for 1-, 2-, 4-, 6-, and 10-wk post-surgery. Two repeat time-course studies were performed for 2-, 4-, and 10-wk and 1-, 2-, and 10-wk post-surgery, respectively. Each of the study groups had six animals for each time point.

### RNA collection

The surgical (right) and contra-lateral (left) tibiae from each group (Sham, ACLT, ACLT+MMx) were rapidly dissected from animals at 1, 2, 4, 6, and 10-wk post-surgery. Tibiae were thoroughly cleaned to remove muscle and ligament as well as joint capsule, without causing undue damage to the tibia plateau. Each tibia was then mounted in a precision bone saw such that the medial side of the bone was oriented parallel to the holding clamp, while the head of the bone was oriented perpendicular to the clamp. Using an Isomet low speed bone saw (Beuhler, Lake Bluff, IL) three cuts were made from the end of each tibia. Slice #1: was approximately 960 μm from the tibial plateau and contained articular cartilage (~200 μm) and subchondral bone (~760 μm). Slice #2: was approximately 1440 μm and contained epiphyseal bone (~1060 μm) and growth plate (~280 μm). Slice #3: was also approximately 1440 μm and contained metaphyseal bone. Each bone slice was placed in saline and flushed with a 23 gauge needle to remove bone marrow. They were quickly frozen in liquid nitrogen and stored at -135°C until processing. The whole process of tissue collection normally took less than 10 minutes from necropsy to freezing of the cartilage and bone slices. Bone slices were pooled for each cut within a group.

### RNA isolation from Bone and Cartilage

Frozen bone slices were immersed in liquid nitrogen and then crushed prior to addition to Trizol reagent (Invitrogen, CA). Samples were homogenized using a Polytron (Brinkman Instruments). Total RNA was isolated according to manufacturer's directions with minor modifications. Following the chloroform extraction step, RNA was re-extracted with acidic phenol:chloroform to remove proteoglycans. RNA was precipitated with isopropanol and washed with 75% ethanol. After pellet was air-dried, RNA was resuspended in molecular biology grade water and concentration of each sample was determined by measuring the absorbance at 260 nm. Agarose gel electrophoresis was used to confirm RNA integrity.

### TaqMan probes and primers

Primers and fluorogenic probes were designed using Primer Express v. 1.0 (Applied Biosystems, CA) and are listed in Table 1. All probes were synthesized by Applied Biosystems with the fluorescent reporter dye FAM (6-carboxy-fluorescein) attached to the 5'-end and the quencher dye TAMRA (6-carboxy-tetramethyl-rhodamine) attached to the 3'-end. When genomic sequence was available, primers and probes were designed to span intron-exon boundaries. Amplified products were designed to be between 70-110 bp.

**Table 1:** Oligonucleotides and TaqMan fluorogenic probes

Gene	Genebank	Primer	Sequence 5'-3'	Amplicon
Aggrecanase-1	NM_023959	Forward	CTGCATCTGCCAGTGACTTTTC	74
		Reverse	TCAGGACCAAAGGTCAGTTGG	
		Probe	TGGCAAGGACTATGATGCTGACCGC	
Bone Sialoprotein	NM_012587	Forward	GTTGGAGTTAGCTGCGCTCC	90
		Reverse	TCCTCTTCCTCGTCGCTTTCCTTCATT	
		Probe	AAGGCTGGAGATGCAGAGGGCAAGG	
Cathepsin K	NM_031560	Forward	GCCATGAATCACCTGGGAGA	76
		Reverse	GCGAAGGTGGCACTCTGAGT	
		Probe	TGACCAGCGAAGAAGTGGTTCAGAAGATGA	
Cbfa-1/Runx2	AF325502	Forward	TGCTTCATTCGCCTCACAA	71
		Reverse	CTTGCTGTCCTCCTGGAGAAA	
		Probe	AACCACAGAACCACAAGTGCGGTGC	
CD31	NM_031591	Forward	TCAACAGAGCCAGCATTGTGA	70
		Reverse	CACGGAGCAAGAAAGACTCTGA	
		Probe	CAGTCTCCGAAGCGGCCCTCTAACA	
Collagen Type IIA	NM_031163	Forward	TCTGCAGAATGGGCAGAGGTATA	103
		Reverse	GATAATGTCATCGCAGAGGACATTC	
		Probe	AAGCCCTCATCTTGCCGCATCTGTG	
Cyclophilin A	NM_017101	Forward	CAAATGCTGGACCAAACACAA	70
		Reverse	GCCATCCAGCCACTCAGTCT	
		Probe	TGGTTCCCAGTTTTTTATCTGCACTGCC	
KDR	NM_013062	Forward	ACGTTTGAGAACCTCACGTGG	84
		Reverse	CTTGCAAACTGGTGTGAGTGATTC	
		Probe	AGCTTGGCTCACAGGCAACATCGG	
MMP-13	NM_133530	Forward	AGTCCTTTTGGCCAGAACTTCC	76
		Reverse	AAGATGAACATGAGGTCTCGGG	
		Probe	CCATGTGGATGCTGCATACGAGCATC	
Osterix	NM_181374	Forward	TGACTGCCTGCCTAGTGTCTACA	94
		Reverse	ACCTGGTGAGATGCCTGCA	
		Probe	ATGTCCCATCCCTACGGCTCCTGGTA	
Osteopontin	NM_012881	Forward	TCTGATGAACAGTATCCCGATGC	84
		Reverse	GACCTTGATAGCCTCATCGGAC	
		Probe	AGGACCTCACCTCCCGCATGAAGAG	
Sox-9	AB073720	Forward	CGCAGGAAGCTGGCAGAC	76
		Reverse	GTCTCCAGAGCTTGCCCAGA	
		Probe	CCGCATCTGCACAACGCGGA	
TRAP	NM_019144	Forward	AATTGCCTACTCCAAGATCTCCAA	74
		Reverse	GCGGAACTTTGAAACGCAAA	
		Probe	CGCTGGAACTTCCCCAGCCCTTATTA	
VEGF	NM_031836	Forward	AGCCCATGAAGTGGTGAAGTTC	71
		Reverse	CCACCAGGGTCTCAATTGGA	
		Probe	TGGACGTCTACCAGCGCAGCTATTGC	

^Exon/intron boundaries are indicated with underlined bases. Exon/intron boundaries for Cathepsin K, TRAP, MMP-9, and VEGF are assumed by comparison to mouse sequence.^

All primer/probe pairs were checked for efficiency of amplification using reverse transcribed rat bone total RNA. Standard curves were generated using serial dilutions of known quantities of total RNA in duplicate.

### Reverse transcription and TaqMan real-time quantitative PCR

Reverse transcription (RT) reactions were carried out for each RNA sample in MicroAmp reaction tubes using TaqMan reverse transcription reagents. Each reaction tube contained 250 ng of total RNA in a volume of 50 μL containing 1× TaqMan RT buffer, 5.5 mM MgCl_2_, 500 μM of each dNTP, 2.5 μM of oligo-d(T)16 primers, 2.5 μM of random hexamers, 0.4 U/μL RNase inhibitor and 1.25 U/μL MultiScribe Reverse Transcriptase. RT reaction was carried out at 25°C for 10 min, 48°C for 30 min and 95°C for 5 min. The RT reaction mixture was then placed at 4°C for immediate use in PCR amplification or stored at -20°C.

Real-time PCR was performed in a MicroAmp Optical 96-well reaction plate. For each 50 μL reaction, 10 μL of RT product (50 ng total RNA), 100 nM forward primer, 100 nM reverse primer, 200 nM probe and 1× Universal Master Mix (Applied Biosystems) were combined. Amplification conditions were 2 min at 50°C, 10 min at 95°C followed by 40 cycles at 95°C for 15 sec, 60°C for 1 min. All reactions were performed in ABI Prism 7700 Sequence Detection System in duplicate. Data collected represents an average from replicate studies for week 1, 2, 4, and 10 and one study for week 6. Expression levels of mRNA were corrected for expression relative to the housekeeping gene, Cyclophilin A, and were expressed as fold change relative to sham levels.

### Immunohistochemistry

Resected knee joints were fixed with 4% paraformaldehyde (Merck, Darmstadt, Germany) in 0.1 M phosphate buffer solution (pH 7.5) for 24 hours at 4°C. After the fixation, the tissues were rinsed with PBS. Tissues were decalcified in 0.5 M ethylene diamine tetra acetate (EDTA) solution (pH 7.6) with stirring in 4°C, then dehydrated in a graded ethanol series and defatted in chloroform to embed into the paraffin wax (Fisher Scientific, NJ, USA). Normal and OA cartilage specimens were examined by histological analysis and immunohistochemistry.

All tissues from the first time course study were processed for tissue sections and randomly selected from each group (N = 6) for immunohistochemical evaluations. Tissue sections were deparaffinized in xylene, hydrated in graded ethanol, then treated with 500 U/ml testicular hyaluronidase (Sigma, MO) at 37°C for 20 min. Tissue sections were incubated with anti-MMP-13 Ab, a cocktail of anti-MMP-9 polyclonal (Cell Sciences, MA) and anti-cathepsin K (CatK) mAb (Oncogene, CA) or a cocktail of anti-VEGF polyclonal (Neomarkers, CA) and anti-KDR mAb (Accurate chemical & scientific Co., NY). All were incubated overnight at 4°C, as described previously [17]. For MMP-13 immunostaining, after rinsing in PBS with 0.3% Tween 20, sections were incubated with biotin-conjugated anti-rabbit Ab (Vector Laboratories, CA) for 30 min, followed by alkaline phosphatase-conjugated streptavidin for 30 min (Vector Laboratories, CA). These sections were rinsed with PBS, and developed using the fast red substrate system (Dako, CA) for 5 min, counterstained with hematoxylin. Double-labeled immunohistochemical staining for cathepsin K/MMP-9 and VEGF/KDR was performed simultaneously. Briefly, tissue sections were incubated with a cocktail of mouse monoclonal and rabbit polyclonal antibodies, followed by incubation with a cocktail of biotin-conjugated anti-rabbit Ab and HRP conjugated anti mouse antibody (DAKO, CA) for 1 hr, and incubated with alkaline phosphatase-conjugated streptavidin for 30 min (Vector Laboratories, CA). Alkaline phosphatase was colored either blue (VEGF) or red (MMP-9) color with AP blue (Vector Laboratories, CA) or fast red (DAKO, CA) respectively. They were washed twice with PBS for 1 hr, followed by development to brown color using 0.5 mg/ml 3, 3'-diaminobenzidine tetrahydrochloride. For controls, the same procedures were carried out either without primary Ab or with mouse mAb IgG instead of primary antibody.

## Results

### Collection of articular cartilage and subchondral bone from the surgically induced joint instability models in the rats

**Figure 1:**
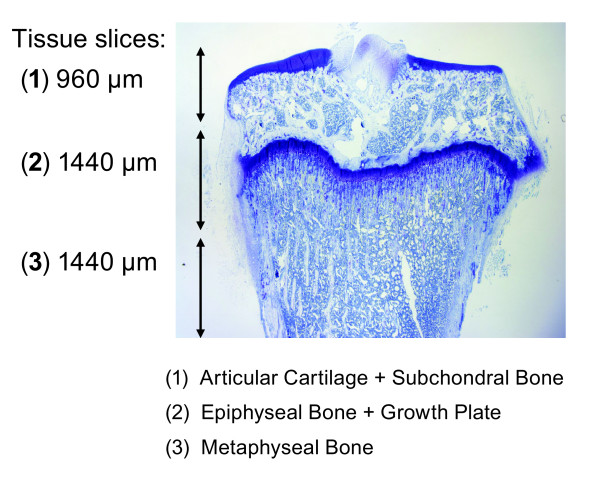
**Schematic representation of tibia sectioning for RNA collection**. Tibiae were serially sectioned using a precision bone saw to obtain bone slices representing discrete areas. Three successive bone slices were prepared according to calibration measurements previously determined from toluidine blue stained sections of aged-matched rodents. The section of primary interest was obtained from the initial slice and included the entire articular cartilage layer and proximal subchondral bone (~760 μm) and measured a total of 960 μm. Slice 2 was predominantly epiphyseal bone and growth plate and measured 1440 μm. The third slice included the adjacent 1440 μm containing metaphyseal bone. Sections two and three were collected as internal controls.

In this study, we initially focused on developing a reliable method for serially collecting the articular cartilage and subchondral bone, the epiphyseal and metaphyseal bone slices from the tibeal plateaux of surgically modified (right) and contra-lateral sham-operated (left) joints for RNA processing. A previous study evaluated time-dependent macroscopic changes in the surgical joint of both OA models in comparison to Sham; detectable changes in the articular cartilage surface at 6 weeks was noted for both ACLT and ACLT + MMx models [17]. To gauge the depth of discrete tissue layers, toluidine blue sections of tibiae from age-matched animals were examined. The resulting measurements were used to calibrate the precision bone saw used for tissue collection for RNA processing. As shown in Figure 1, slice 1 contains the articular cartilage and the proximal subchondral bone layer, collected from the first 960 μm measured from the tibial surface; slice 2 contains mostly the epiphyseal bone and growth plate, spanned the next 1440 μm; and slice 3 contains the metaphyseal bone, collected from the adjacent 1440 μm.

From a study with aged matched rats subjected to either sham-operated, or to anterior cruciate ligament transection (ACLT) alone or transection of both ACL and medial collateral ligament and partial resection of medial meniscus (ACLT+MMx), total RNA extracted from the three tissue slices was found to have high quality and relatively consistent amounts between the collected individual joints. To minimize animal variability, six slices per group were combined for each time point. Total RNA isolated from the first slices containing both articular cartilage and subchondral bone was evaluated by real time PCR using known gene markers for cartilage and bone catabolism and anabolism (Table 2). It should be noted that the real time PCR data represented the averaged values from three independent time-course experiments.

**Table 2:** Summary of gene expression patterns regulated in joint-instability induced osteoarthritis progression in rats (slice#1)

Gene	ACLT					ACLT+MMx				
	**(Fold increase over Sham in articular cartilage/subchondral bone region)**									
**Week post-surgery**	**1**	**2**	**4**	**6**	**10**	**1**	**2**	**4**	**6**	**10**

*Matrix Degradation*										
Aggrecanase I	4.3	2.1	2.5	1.6	2.6	4.2	1.7	2.9	2.3	3.2
MMP13	2.8	1.6	1.9	2.0	4.3	3.7	1.6	2.0	2.2	5.8
*Chondrocyte differentiation*										
Collagen IIA	2.3	2.0	2.8	1.3	1.3	1.8	1.5	2.2	1.4	1.2
Sox 9	2.6	1.8	1.4	1.4	0.8	1.8	1.1	1.4	1.8	1.4
*Angiogenesis*										
VEGF	1.3	2.1	1.6	1.8	1.1	1.7	1.3	1.8	1.3	1.6
CD31	2.1	3.1	1.6	1.8	1.4	2.2	2.0	1.9	1.4	2.3
KDR	1.3	3.2	1.3	1.3	1.1	1.4	2.2	1.3	1.2	1.3
*Bone resorption*										
Cathepsin K	1.5	1.5	1.8	2.7	2.5	1.8	1.9	2.1	2.0	4.1
TRAP										
*Bone formation*										
Runx2/cbfa-1	0.7	1.0	1.1	1.8	1.2	0.9	0.9	1.5	1.8	2.0
Osterix	0.7	0.9	1.0	1.7	1.1	1.2	0.9	1.6	1.6	1.6
Bone sialoprotein	1.1	1.4	1.7	1.8	1.6	1.6	1.3	1.5	1.9	2.9
Osteopontin	1.3	1.3	1.9	1.5	1.8	1.7	1.1	1.8	2.4	4.1

**Figure 2:**
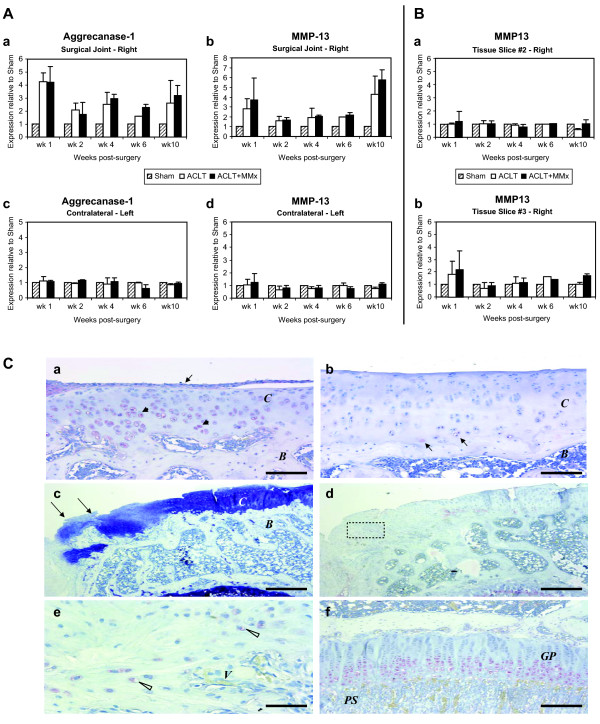
**Up-regulation of matrix degradation markers as an early gene regulation event in the surgically-induced models of osteoarthritis**. (A) Up-regulation of matrix degradation markers was seen as early as one week following surgery. (a) Aggrecanase-1 demonstrated early increases of 3-4 fold in ACLT+MMx and ACLT models, respectively. High levels of expression were maintained throughout the 10 wks of study duration. (b) MMP-13 levels were elevated 2-fold at wk 1 post-surgery. Levels progressively increased by 3 to 5-fold relative to Sham through wk 10. (c, d) Expression levels of both markers were unaffected in contra-lateral joints over entire time course. (B) No changes in MMP-13 mRNA expression were observed in deep tissue slice 2 (a) or slice 3 (b). Similar results were observed for aggrecanase-1. All values are shown as mean ± SD. (C) Frontal section of medial tibial plateau and growth plate were stained with anti-MMP-13 (a, b, d, e, and f) or toluidine blue-O (c). Articular cartilage, including growth plate, at wk 2 (a and b) and at wk 10 (c, d, e, and f) post-surgery are shown. (a) ACLT+MMx OA joint highly expressed MMP-13 in differentiated chondrocytes (closed arrowheads) and synovial cells on cartilage surface (arrow). (b) In sham joint, few cells are MMP-13 positive in deep zone (arrows). (c) Arrows indicate osteophyte formation in periarticular region of the medial tibial plateau of ACLT-MMx. (d) MMP-13 immunostaining of semi-serial section of (c). (e) Higher magnification of osteophyte region (inset in d) shows many MMP-13 positive chondrocytes are found in proximity to vascular invasion (open arrowheads). (f) In growth plate, as a positive control, MMP-13 was intensely stained in hypertrophic chondrocytes. *C*: articular cartilage. *B*: bone. *V*: vascular invasion. *GP*: growth plate. PS: primary spongiosa. Bar = 100 μm in (a, b), 500 μm (c, d), 50 μm (e), 200 μm (f).

In articular cartilage and subchondral bone slices (slice#1), there were time-dependent and marked changes in the expression profiles of known cartilage degeneration genes in response to either ACLT or ACLT+MMx surgically induced joint instability, as compared to those in the contra-lateral left joints and Sham-operated joints (Figure 2A). However, there were only subtle differences in the profiles of these cartilage and bone makers in the epiphyseal (slice#2) and metaphyseal (slice#3) slices in the ACLT or ACLT+MMx joints as compared to the contra-lateral joints (Figure 2B and data not shown). Therefore, in this study, we focused further analyses in slice 1 to study regulation of key markers (Table 2) for cartilage matrix degradation (aggrecanase-1, metalloproteinase-13 (MMP-13)), chondrocyte differentiation (collagen IIA and Sox 9), angiogenesis (VEGF, CD31, KDR), bone resorption (cathepsin K and tartrate resistant acid phosphatase (TRAP)) and bone formation (runx2, osterix, bone sialoprotein and osteopontin).

### Early regulation of markers for matrix degradation in the articular cartilage and subchondral bone in the rat models of joint instability-induced osteoarthritis

The gene expression patterns during disease progression in the ACLT and ACLT+MMx models of joint instability versus Sham-operated animals were characterized by mRNA levels using real time PCR and confirmed by protein levels using immunohistochemical methods. Early changes evident in OA include surface cartilage damage and subchondral bone remodeling. As shown in Figure 2A, aggrecanase-1 and MMP-13, proteolytic enzymes which degrade the cartilage matrix, were up-regulated as early as week 1 in both OA models as compared to Sham. Expression levels of aggrecanase-1 aggressively increased 3-4 fold in both ACLT+MMx and ACLT models, respectively, relative to Sham (Figure 2A-a; Table 2). The aggrecanase-1 mRNA expression levels remained elevated at a high steady-state above Sham up to 10-week post-surgery, supporting the relevant role of this enzyme in cartilage degeneration during the initial and progressive phases of OA.

The regulation of MMP-13 synthesis was similar, yet more modest, to that of aggrecanase-1 in ACLT and ACLT+MMx joints. MMP13 expression levels in the transected joints were elevated by ~2- fold as early as week 1, relative to Sham and remained elevated relative to Sham peaking at 3-5 fold by week 10 (Figure 2A-b; Table 2). Remarkably, regulation of these two enzyme markers was specific to the diseased joint as their respective levels were not different in contra-lateral intact joints (left), relative to Sham-operated joints (Figure 2A-c, 2d). Furthermore, there were no changes in aggrecanase-1 (data not shown) or in MMP-13 mRNA levels (Figure 2B-a, 2b) detected in deeper epiphyseal and metaphyseal bone tissue layers (slices 2 & 3) in any treatment group.

Immunohistochemical staining of the frontal section of the medial tibeal plateau and growth plate demonstrated sustained MMP-13 expression as shown in Figure 2C. In the ACLT + MMx model, protein levels of MMP-13 were apparent in the differentiated chondrocytes and synovial cells of the cartilage surface (Figure 2C-a) at 2 weeks following surgery. There was low, but detectable MMP-13 protein levels evident in the Sham articular cartilage, i.e. in a few cells in the deep zone (Figure 2C-b). At week 10 post-surgery, significant cartilage thinning and fraying, accompanied with advancement of subchondral bone and osteophyte formation (Figure 2C-c, arrowheads) were readily detected as a late stage event of OA progression. Osteochondral progenitors within the osteophyte region showed high levels of MMP-13 protein expression (Figure 2C-d with inset and Figure 2C-e, shown at high magnification of inset) within proximity to areas of vascular invasion. As a positive control, we stained for MMP-13 proteins known to be highly expressed in hypertrophic chondrocytes in the epiphyseal bone and growth plate (slice 2) of the ACLT+MMx joints in rats (Figure 2C-f). Similar to the results obtained from the real time PCR study, MMP-13 expression levels although expressed in high abundance, were not regulated in tissue slice 2 in response to ACLT or ACLT+MMx as compared to Sham or contra-lateral intact joints (Figure 2B).

### Temporal regulation of gene markers for chondrocyte differentiation in the rat models of osteoarthritis

**Figure 3:**
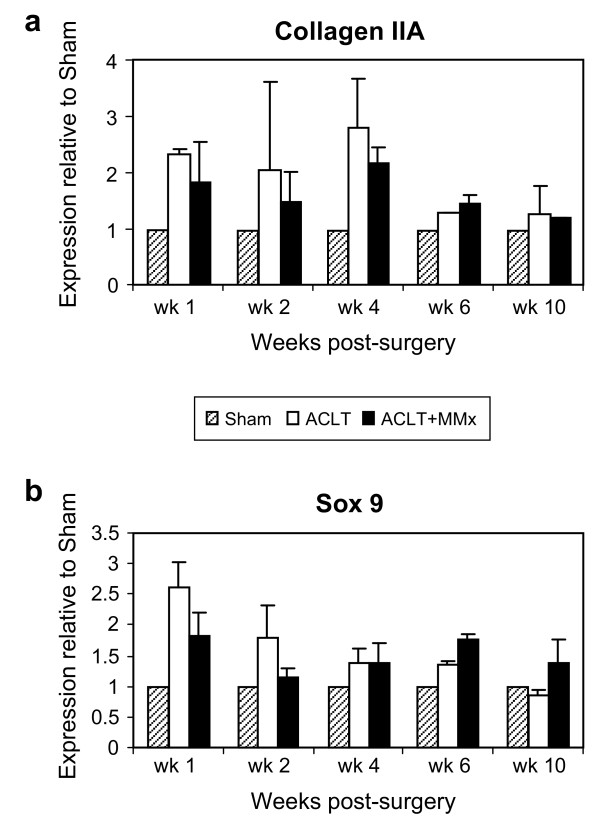
**Temporal regulation of markers of chondrocyte differentiation in the surgically-induced models of OA**. (A) Collagen IIA was increased by 2-fold in ACLT model at wk 1 relative to Sham. Expression levels peaked at 3-fold through wk 2 and 4 and returned to Sham levels in wk 6 and 10. (B) Sox 9 demonstrated a similar, those less striking pattern of expression. Sox 9 was upregulated at wk 1 in ACLT and returned to Sham levels in wk 6 and 10. The fold changes in Col IIA levels were significantly lower in ACLT+MMx as compared to those in ACLT model. All values are shown as mean ± SD.

Evidence has been presented suggesting that phenotypic alteration in articular chondrocytes is a facet of disease progression in OA [18]. Chondrocyte differentiation has been demonstrated to be an early event in disease progression with increased expression of collagen IIA (Col IIA), a splice variant normally expressed in chondroprogenitor cells during embryonic development [19]. Here, the ACLT model showed a 2-fold increase in Col IIA, relative to Sham at week 1, peaked at 3-fold through weeks 2 and 4 and returned to Sham level in week 6 to 10 (Figure 3A; Table 2). Upregulation of Col IIA was also seen in the ACLT+MMx model, rising steadily to a maximum of 2-fold at 4-week post-surgery, and subsequently returned to Sham levels during the later stage of disease progression (Figure 3A; Table 2). We also investigated the temporal regulation of Sox 9, a marker for chondrocyte differentiation. Similar to the Col IIA expression pattern, Sox 9 expression was upregulated in ACLT-joints as early as week 1, followed by a progressive return to baseline levels by week 10 (Figure 3B; Table 2). Although the ACLT+MMx model has been characterized to have more aggressive disease progression, the levels of Col IIA and Sox 9 regulation were lower than those in the ACLT model at weeks 1 and 2 (Figure 3B, Table 2).

### Temporal regulation of gene markers for angiogenesis in the rat models of osteoarthritis

**Figure 4:**
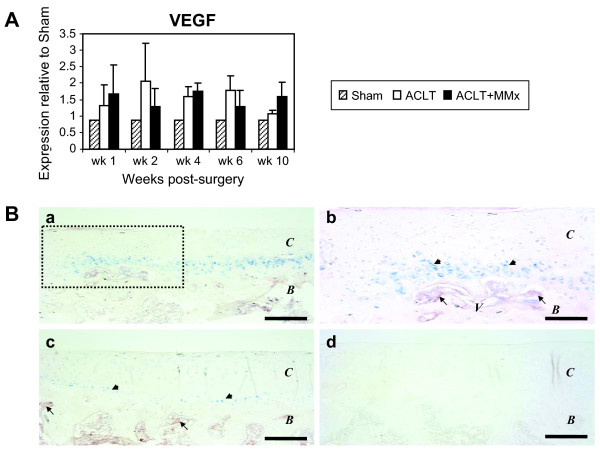
**Up-regulation of markers of angiogenesis in the surgically-induced models of joint instability**. (A) Induction of angiogenesis gene expression marker, VEGF, was seen at 2-wk post-surgery in both models. Levels of VEGF increased approximately 2 fold at week 2, maintained expression during wk 4 and 6 and returned to Sham levels by week 10. All values are shown as mean ± SD. (B) Frontal section of medial tibial plateau and growth plate were stained with anti-VEGF (blue) and anti-KDR (brown). (a) At 2-wk post-surgery, many chondrocytes differentiated to hypertrophic chondrocytes in ACLT+MMx joint. (b) Higher magnification of inset in (a) showed that hypertrophic chondrocyte-like cells strongly stained with VEGF (arrowheads). KDR (brown) positive cells are found in proximity with VEGF-positive cells (arrow). (c) Sham joint shows a few VEGF-positive cells in deep zone of articular cartilage (arrowheads). Vascular endothelial cells are positive for KDR in bone marrow, but not in proximity with VEGF-positive cells (arrows). (d) Negative control shows no specific staining. *C*: articular cartilage. *B*: bone. *V*: vascular invasion. Bar = 200 μm in (a, c and d); and 100 μm in (b).

Vascular invasion into calcified cartilage is obligatory in normal endochondral bone formation and has also been demonstrated to occur during disease progression in experimental models of OA. We examined the time-dependent regulation of typical gene markers for angiogenesis in tissue slice #1, containing articular cartilage and subchondral bone. The angiogenesis marker, VEGF, was up-regulated approximately 2 fold at week 2 and week 4, in ACLT and ACLT-MM respectively, responding to joint instability in both surgically-induced models, (Figure 4A; Table 2). Expression remained elevated relative to Sham in both models through week 6. As shown in Table 2, we also examined the regulation profiles of two other angiogenic markers including CD31 and the VEGF receptor, KDR. Similar to the regulation pattern of VEGF, both CD31 and KDR were highly upregulated at week 2, by ~3-fold in the ACLT joints and by ~2-fold in the ACLT+MMx joints (Table 2). Expression of these two vascular markers returned towards Sham levels by week 10 in the ACLT joints, yet remained slightly elevated above Sham levels up to week 10 post-surgery (Table 2). in ACLT-MMx joints.

Vascular invasion as an early event in ACLT and ACLT+MMx joints was further confirmed using immunohistochemistry. From representative thin sections isolated from an ACLT+MMx joint at week 2 post-surgery, the number of VEGF-positive (blue) hypertrophic chondrocyte-like cells significantly increased in the deep articular cartilage layer, adjacent to subchondral bone (arrowheads, Figure 4B-a, and inset shown in b). In proximity to the VEGF-positive chondrocytes, we detected vascularized structures positive for KDR (brown) invading the calcified deep cartilage layer (arrows, Figure 4B-b). In the Sham joint, very few VEGF-positive stained cells were detected in the deep zone of articular cartilage (arrow-heads, Figure 4B-c). However, KDR-positive signals could be readily detected in vascular endothelial cells in the bone marrow of the epiphysis, distant from VEGF (arrows, Figure 4B-c).

### Progressive increases in gene markers for bone remodeling in the rat models of osteoarthritis

**Figure 5:**
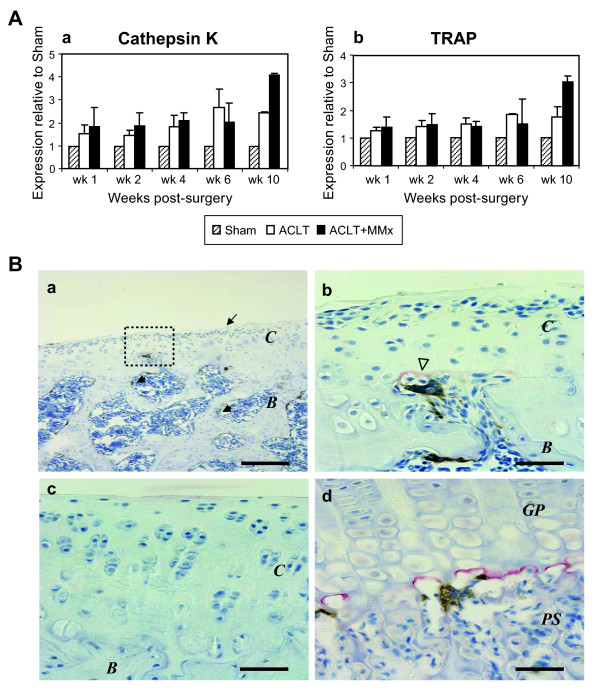
**Bone resorption markers are increased in rat models of OA**. (A) Up-regulated expression of Cat K and TRAP (a) Cat K levels progressively increased by 2 to 4-fold in ACLT and ACLT-MMx, respectively, versus Sham. (b) Similarly, time-dependent upregulation of TRAP expression peaked at 2 to 3 fold in ACLT and ACLT-MMx, respectively. All values are shown as mean ± SD. (B) Immunolocalization of osteoclastic markers (MMP-9 and Cat K) in ACLT-MMx joint at 2-wk after surgery. (a-d) Frontal section of medial tibial plateau and growth plate were stained with anti-Cat K (brown) and anti-MMP-9 (red). (a) At 2-wk post-surgery, irregular articular surface is observed (arrow). Cat K positive multinucleated cells detected on trabecular bone surface in bone marrow (arrowheads). (b) Cat K positive cells invading into articular cartilage are found in proximity to MMP-9 (red) staining (open arrowhead) with higher magnification of squared area in (a). (c) Negative control shows no specific staining. (d) Growth plate and primary spongiosa, stained as positive control, demonstrated Cat K positive cells in proximity to MMP-9 stained region in the border of cartilage and primary spongiosa. *C*: articular cartilage. *B*: bone. *GP*: growth plate. PS: primary spongiosa. Bar = 500 μm in (a, c); 50 μm in (b, d).

In addition to cartilage degeneration, subchondral bone remodeling and osteophytosis are notable events during the disease progression in both of the surgically-induced models in rats. We previously documented early involvement of subchondral osteopenia, which was followed by subchondral sclerosis in response to joint instability in both ACLT and ACLT+MMx models of OA in rats. Hence, time dependent regulation of subchondral bone loss was assessed by two different gene markers specific for osteoclastic bone resorption, CatK and tartrate resistant acid phosphatase (TRAP) using real-time PCR of tissue slice #1. Expression of mRNA levels of CatK was elevated (1.5 to 2-fold) as early as week 2 post-surgery, then steadily increased to maximal expression (~2 to 4-fold) at week 10 in the ACLT and ACLT+MMx models, respectively (Figure 5A-a). The pattern of time dependent regulation of TRAP was similar to that of CatK (Figure 5A-b), reconfirming these are selective markers for mature osteoclasts.

The levels of CatK (brown) and MMP-9 (red) were co-stained using immunohistochemical methods with paraffin embedded sections from typical ACLT+MMx joints versus Sham joints at week-2 post-surgery (Figure 5B). At 2 weeks post surgery irregular articular surface was observed in the OA-joints (arrow, Figure 5B-a). In the articular cartilage and subchondral regions, CatK expression was detected in multinucleated osteoclast-like cells invading into the deep calcified cartilage layer as an early event in this surgically-induced model (arrowheads, Figure 5B-a, and inset shown in b). Interestingly, MMP-9-positive signal (red) was only localized in the extracellular matrix adjacent to hypertrophic chondrocytes specifically in proximity to the invading osteoclasts (open arrowhead, Figure 5B-b). There was no MMP-9 expression detected in the vicinity of the multinucleated osteoclasts on subchondral bone surfaces (arrowheads, Figure 5B-a). A similar pattern of MMP-9 expression in hypertrophic chondrocytes in proximity to osteoclasts was found at the border of the growth plate and the mineralized primary spongiosa (Figure 5B-d). As control, CatK and MMP-9 were localized to osteoclasts in the subchondral bone plate of the Sham-joints. No detectable CatK/MMP-9 positive cells were observed to have invaded the Sham articular cartilage (data not shown).

**Figure 6:**
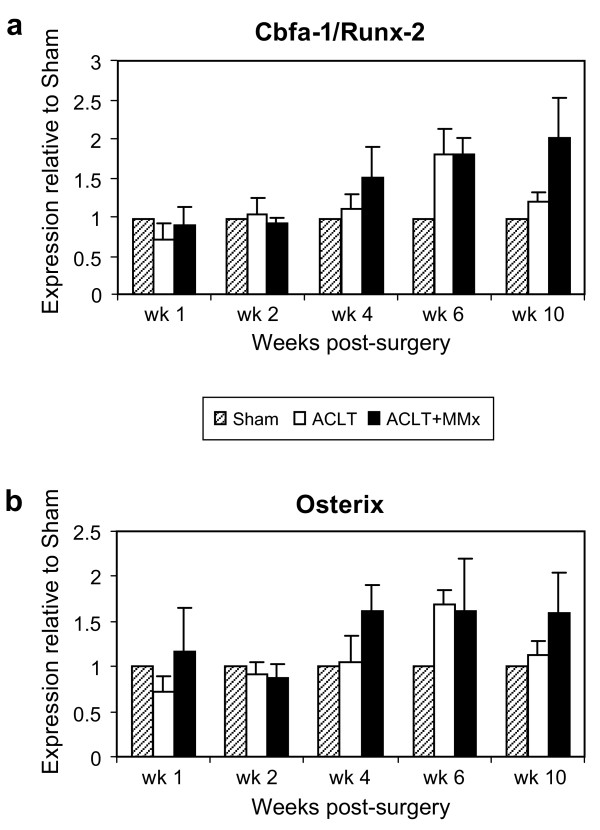
**Up-regulation of transcription factors mediating bone anabolism, runx2 and osterix in the rat surgical models of OA**. Bone formation marker runx2 (a) and its downstream target osterix (b) were up-regulated during the later stage of disease progression in ACLT+MMx by ~ 2-fold in wk 4 post-surgery, and in ACLT by ~ 2-fold in wk 6 post-surgery relative to Sham. All values are shown as mean ± SD.

Subchondral sclerosis and osteophyte formation occur late in the disease progression in these experimental models of OA. Two transcription factors, Runx2/Cbfa1 and osterix, have been shown to play critical roles in mediating bone anabolism. Runx2 plays the rate-limiting role in regulating bone formation, and also regulates osterix during osteoblast differentiation [20]. Neither runx2 nor osterix increased until the late stages (up to 2-fold at week 4 to 6) of disease progression in ACLT+MMx and ACLT models, respectively (Figure 6-a, b, Table-2). Similar regulation patterns were also seen with bone sialoprotein and osteopontin in these surgically-induced models of OA (Table 2).

## Discussion

Accumulating evidence supports the hypothesis that subchondral bone remodeling in OA is a major contributing factor to this degenerative disease [21]. The integrity of articular cartilage depends on the mechanical as well as biochemical properties of the underlying bone [21,22]. In spontaneous OA animal models and in OA patients, density and metabolism of the subchondral bone have been suggested to occur prior to detectable signs of cartilage damage [22-26]. We have previously shown in the ACLT and ACLT+MMx models of OA in rats, histological evidence demonstrating that joint instability also accelerated subchondral remodeling, occurring concomitantly and in proximity to surface cartilage damage in early stage OA [16]. As the disease advances, in OA animal models significant cartilage thinning, subchondral bone sclerosis and osteophyte formation has been observed [15,27-29].

To understand the molecular mechanisms of cartilage degeneration and subchondral bone sclerosis and the potentially coordinated signals between the two tissues during OA progression, we developed a fast and uniform approach to collect together the articular cartilage and subchondral bone tissues from the tibia of the ACLT and ACLT+MMx models of OA in rats. Prior to performing extensive genome-wide profiling of gene expression and pathway analyses in these models, we described here the validation of this approach by demonstrating the time-dependent and sequential changes in these tissues using real-time PCR and confirmation with immunohistochemical methods for genes previously demonstrated to be involved in the pathogenesis of OA.

Previous studies have evaluated gene expression *in vivo *either by means of global profiling, or by studying subsets of collagenases or cytokines in articular cartilage in similar experimental models of OA [30-33]. These studies demonstrated that the gene expression changes during the disease progression in animal models of OA mimic that in human. Experimental models of OA provided important insights on key signaling pathway(s) that involved proteases, cytokines or growth factors in mediating OA pathogenesis. However, these early studies were primarily restricted to examining molecular changes in the cartilage layer, without integrating information from the pre-articular subchondral bone in response to the surgically-induced joint instability. Furthermore, these studies employed collection of articular cartilage from the small joints of the rodent [34,35], by pooling pieces of cartilage tissues shaved from the bone surface. This method often results in high variability in the amount and quality of RNA collected from the joints. We sought to reduce this variability and investigate bone in addition to cartilage by systematically collecting the tibial plateau slice containing both articular cartilage and the underlying subchondral bone. Variability was further reduced by combining slices from each cut within a group, and performing replicate time-course studies. Expression levels of each RNA pool were compared to expression levels from sham operated joints for each independent study increasing the confidence of gene expression changes demonstrated. It should be noted that this does limit the ability to detect animal specific changes within a group. Also, inherent variability in biological models is reflected in the degree of fold change demonstrated, thus error bars are large in some instances although the direction of change was always consistent. The reduction in the time of tissue collection by using serially collected bone slices yields an additional advantage. Thus, this method has potential application for high-throughput collection of tibial plateaux tissues involved in OA pathogenesis.

While our method has the advantage of consistently sampling RNA for a global assessment of the joint tissues, we recognize the approaches limitation to evaluate the specific changes in bone or articular cartilage, due to the common expression of many genes in both chondrocytes and osteoblasts. On the other hand, to support the long-term genome-wide profiling efforts to increase our understanding of the pathogenesis of the degenerative joints in response to mechanical instability, we have collected the combined subchondral bone and articular cartilage from the spontaneous STR/ort mice [36] and the surgically induced rat models. In addition, we have also collected separately the articular cartilage layer and the subchondral bone from the ACLT-dogs and from the humans with primary OA for array studies. By integrating information for gene pathway analyses on these tissues from multiple species, we believe this will provide a comprehensive approach to identify novel mechanism(s) involved at multiple tissue levels during OA progression.

Early degenerative changes have been reported in the superficial zone of articular cartilage in both models and consisted of proteoglycan loss and detectable surface collagen damage, accompanied by increased chondrocyte proliferation and hypertrophy within 2 weeks of surgery [16,31,37,38]. Preceding the histological changes in the ACLT and ACLT+MMx joints, we showed that the expression patterns of MMP-13 and aggrecanase-1, responsible for degradation of collagen type II and of proteoglycan core proteins, respectively, were quickly and significantly up-regulated as early as the first week post-surgery. Interestingly, expression of these proteases remained significantly elevated above basal levels during the 10 week study, confirming the important roles of these enzymes in the continuous break down of tissues within the joint as the disease progresses [39-41].

The onset of chondrogenic differentiation has been confirmed by the appearance of glycosaminoglycan, aggrecan and Col IIA, a splice variant of the Col II gene, expression by chondroprogenitors in the extracellular matrix [42]. The transcription factor Sox 9 is required for mesenchymal condensation. Together with Sox 5 and Sox 6, Sox 9 mediates chondrocyte differentiation and expression of a series of chondrocyte-specific marker genes including Col2a1, Col9a2, Col11a2 and aggrecan [43]. Activation of Sox 9 is also important for endochondral bone formation. In our hands, within the first week post-surgery, gene markers for chondrocyte differentiation, Col IIA and Sox 9, were highly upregulated. However, unlike the markers of matrix degradation, expression levels of Col IIA declined by week 6 post-surgery, while Sox 9 was significantly reduced by week 2 post-surgery. This finding may support the hypothesis that early chondrocyte differentiation in response to joint instability is an attempt to repair the tissue during the early phase of disease progression [19]. Mesenchymal condensation and chondrocyte differentiation have been implicated in the early stages of osteophyte formation [44] and potentially, this upregulation of Sox 9 and Col IIA may reflect the initial commitment of the osteochondral progenitors to the development of osteophytes in ACLT and ACLT+MMx models.

Normal adult articular cartilage remains avascular. Vascularization of the articular cartilage, preceding subchondral remodeling and osteophytosis, is a hallmark of the pathology of OA [45]. Previously, in the ACLT and ACLT+MMx model, vascular invasion into the calcified deep layer of articular cartilage could be detected as early as 2 weeks post-surgery [16]. Here, significantly increased expression of the angiogenic factor VEGF was detected at week 2 and remained elevated up to week 6 post-surgery in both models as compared to Sham. Similar to VEGF, the expression profile of its receptor, KDR, and the endothelial marker, CD31, were highly upregulated at 2 weeks post-surgery. Interestingly, in the osteochondral junction of the surgically-induced joints, the CD31-positive blood vessels were frequently found to be in proximity of the chondrocytes expressing VEGF, suggesting early vascular invasion into cartilage may lead to focal endochondral bone formation, and subsequently to subchondral bone remodeling. This process may eventually promote osteophytosis at the late stage of disease progression. All immunohistochemical observations are consistent with our previous histological survey on vascularization in these rat models of OA [17].

As determined by histological evaluation, vascular invasion into calcified cartilage at the osteochondral junction was almost always found together with the presence of osteoclasts, and preceded subchondral bone sclerosis. We have reported previously in the ACLT and ACLT+MMx models of OA, that subchondral osteopenia was significant ~2 weeks post-surgery, soon after the initially observed focal surface cartilage damage and in absence of marked cartilage thinning [16]. Indeed, the bisphosphonate alendronate was demonstrated to retard the disease progression in the ACLT model of OA in rats [46]. Even though multinucleated osteoclasts represent a very small fraction of the total cellular contents in cartilage and bone tissues, upregulation (1.5 to 2-fold) of osteoclast-specific markers, CatK and TRAP, was readily detected in ACLT and ACLT+MMx joints as early as 2 weeks post-surgery. This observation coupled to subsequent subchondral bone remodeling may explain the progressive elevation of these two markers in the articular cartilage and subchondral bone slice in the OA joints.

Changes in subchondral bone are believed to occur in the later stage of OA, particularly osteophytosis. While runx2/cbfa-1 is also expressed in hypertrophic chondrocytes, this transcription factor is the master gene of bone formation [47]. Osterix is a runx2 dependent osteoblast transcription factor, required for bone formation. Expression of runx2 and osterix together is fundamental for mesenchymal commitment to osteogenesis. In addition to runx2 and osterix, we also determined the regulation of two preferential matrix markers for osteoblasts, bone sialoprotein and osteopontin. We demonstrated that regulation profiles of the transcription factors runx2 and osterix, as well as osteogenic matrix proteins, were initiated at week 4 and maximal by 6 week post-surgery; thus supporting previous histological findings demonstrating a significant increase in subchondral bone sclerosis and osteophyte formation toward the later stages of disease progression in these two surgical models of OA [16]. Different from other markers associated with cartilage changes, the degree of change of the bone remodeling genes appeared to be more severe in the ACLT+MMx relative to that in the ACLT model. This is in agreement with previous histological findings [16] where osteophyte development in ACLT+MMx model was found to be more severe than that in ACLT model at week 6 and 10 post-surgery.

## Conclusion

In summary, articular degeneration and bone sclerosis are the most consistently observed characteristics of osteoarthritis (OA). The surgically-induced joint instability models of OA in rats, which resemble post-traumatic OA in humans, are useful tools to study OA pathogenesis. To evaluate the potential coordinating molecular mechanisms of cartilage degeneration and subchondral bone remodeling during disease progression, we developed a consistent and reliable method to harvest both articular cartilage and subchondral bone from the tibial plateaux of the surgically-induced joint instability models of OA in rats. Here, we described the validation of this approach by demonstrating the time-dependent and sequential changes in gene expression profiles of key markers for cartilage degradation, chondrocyte differentiation, vascular invasion and bone remodeling in these tissues using real-time PCR and confirmation with immunohistochemical methods. From the experimental models and humans with OA, rich sources of published information on the key pathways regulated in the articular cartilage and synovium are available [48,49]. However, the same type of information for the subchondral bone is still lacking. To gain further understanding of the sequential and cohesive regulation of complex and potential novel biological pathways in the joints during disease progression, the findings in this study have focused our future efforts on genome-wide profiling of gene expression and pathway analyses of the subchondral cartilage and bone. Integrated information on gene profiling of the subchondral bone during OA progression will be analyzed by cross referencing the results from the combined tissues to existing published articular cartilage databases.

## Competing interests

Maureen Pickarski, Ya Zhuo, and Le T. Duong are employees of Merck, the studies' sponsor, and may own stock/stock options in the company. Tadashi Hayami was an employee of Merck during the conduct of these studies and the preparation of this manuscript.

## Authors' contributions

MP, TH and LD designed and executed the animal study design, in vivo OA surgery, the development of tissue collection methods and the study necropsy. MP carried out all RNA collection, real-time PCR experiments and subsequent analyses. TH and YZ carried out immunohistochemical methods. MP, TH and LD helped to draft the manuscript. All authors read and approved the final manuscript.

## Pre-publication history

The pre-publication history for this paper can be accessed here:

http://www.biomedcentral.com/1471-2474/12/197/prepub
